# The pseudo-Michael reaction of 1-aryl-4,5-dihydro-1*H*-imidazol-2-amines with ethyl ethoxymethylenecyanoacetate

**DOI:** 10.1007/s00706-013-0982-y

**Published:** 2013-05-14

**Authors:** Agnieszka A. Kaczor, Urszula Kijkowska-Murak, Kalevi Pihlaja, Jari Sinkkonen, Waldemar Wysocki, Zbigniew Karczmarzyk, Dariusz Matosiuk

**Affiliations:** 1 Department of Synthesis and Chemical Technology of Pharmaceutical Substances with Computer Modeling Lab, Faculty of Pharmacy with Division of Medical Analytics, Medical University of Lublin, 4A Chodźki St, 20093 Lublin, Poland; 2 Department of Chemistry, University of Turku, Vatselankatu 2, 20014 Turku, Finland; 3 Department of Chemistry, University of Podlasie, 3 Maja 54 St, 08110 Siedlce, Poland

**Keywords:** Annelation, Michael addition, X-ray structure determination

## Abstract

**Abstract:**

The pseudo-Michael reaction of 1-aryl-4,5-dihydro-1*H*-imidazol-2-amines with ethyl 2-cyano-3-methoxyprop-2-enoate (ethyl ethoxymethylenecyanoacetate) is investigated. At −10 °C reaction takes place on the exocyclic nitrogen atom, giving exclusively ethyl esters of 2-cyano-3-[(1-phenyl-4,5-dihydro-1*H*-imidazol-2-yl)amino]prop-2-enoic acid. The formation of isomeric enamines which may be a theoretical product of the reaction on N3 ring nitrogen atom is not observed. The N6 enamines, heated in boiling acetic acid, yield cyclic 1-aryl-5-oxo-2,3-dihydroimidazo[1,2-*a*]pyrimidine-6-carbonitriles. Heating of the enamines to the temperature of 120–140 °C without a solvent makes it possible to obtain a mixture of 1-aryl-5-oxo-2,3-dihydroimidazo[1,2-*a*]pyrimidine-6-carbonitriles and ethyl 1-aryl-5-imino-2,3-dihydroimidazo[1,2-*a*]pyrimidine-6-carboxylates. The reaction of the respective hydrobromides of 1-aryl-4,5-dihydro-1*H*-imidazol-2-amines with ethyl ethoxymethylenecyanoacetate in the presence of triethylamine gives selectively 1-aryl-5-oxo-1,2,3,5-dihydroimidazo[1,2-*a*]pyrimidine-6-carbonitriles.

**Graphical Abstract:**

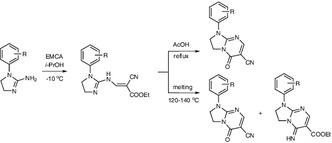

## Introduction

Ethyl 2-cyano-3-methoxyprop-2-enoate (ethyl ethoxymethylenecyanoacetate, EMCA, **1**) (Scheme 1) is a widely used reagent for heterocyclic annelation [1]. In particular, EMCA is applied as the Michael acceptor in Michael addition [1]. Reaction of EMCA with 4,5-dihydro-1*H*-imidazol-2-amines leads to 2,3-dihydroimidazo[1,2-*a*]pyrimidines [1–4] and represents one of the synthetic methods of this heterocyclic system by reaction of imidazol-2-amine derivatives with electrophilic compounds (the main alternative involves the imidazole ring closure by condensation of pyrimidin-2-amines with an appropriate compound) [5]. The imidazo[1,2-*a*]pyrimidine system is present in many biologically active compounds which have been reported to exhibit anti-inflammatory and analgesic [6–9], antibacterial [10–14], antiviral [15], antifungal [16, 17], insectidal, acaricidal, and nematocidal [18], central nervous system (CNS) [19–23], hormonal [24], mutagenic [25], anticancer [26, 27], and cardiovascular [28] activity.

**Figure:**
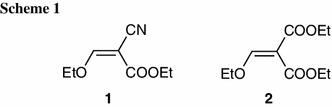

We have previously reported the pseudo-Michael reaction of 4,5-dihydro-1*H*-imidazol-2-amines with diethyl (methoxymethylidene)propanedioate (diethyl ethoxymethylenemalonate, DEEM, **2**) (Scheme 1) [29]. The isolation of chain enamines was not possible for the adducts with DEEM [29] which is in agreement with corresponding literature data (for review see [1]). In the case of EMCA it is relatively easy to isolate the adducts which are the direct products of Michael reaction [1]. Such adducts may possess the EMCA part connected with nitrogen (enamines) [30–32], carbon [33], and sulfur [34] atoms and may be further cyclized via ester [1, 35, 36] or cyano [1, 37, 38] group.

In this paper we present the pseudo-Michael reaction of 4,5-dihydro-1*H*-imidazol-2-amines with EMCA and structural studies of the respective products.

## Results and discussion

The hydrobromides **3a**–**3f** of 4,5-dihydro-1*H*-imidazol-2-amines **4a**–**4f** were obtained from the respective *N*-aryl-1,2-diaminoethanes and cyanogen bromide, as previously reported [29, 39, 40]. The hydrobromides **3a**–**3f** were then transformed into free bases **4a**–**4f** by action of sodium hydroxide and extraction with methylene chloride. The 4,5-dihydro-1*H*-imidazol-2-amines **4a**–**4f** were subjected to the pseudo-Michael reaction with EMCA in propan-2-ol solution at −10 °C (Scheme 2). In these conditions the only isolated products were chain enamines (**5a**, **5c**–**5h**), formed as a result of the reaction on the N6 exocyclic nitrogen atom. The attempt to obtain derivative **5b** with 2-chloro substituent failed, probably due to the steric hindrance. The reaction on the N6 nitrogen atom was in contrast to our earlier results on the pseudo-Michael reaction of 4,5-dihydro-1*H*-imidazol-2-amines with DEEM [29]. In the case of DEEM, at −10 °C reaction took place on the N3 ring nitrogen atom, but the isolation of the respective chain enamines was not possible due to the fast cyclization process, even at low temperature. Instead, ethyl 1-aryl-7-oxo-2,3-dihydroimidazo[1,2-*a*]pyrimidine-6-carboxylates were obtained exclusively. As was found previously, the pseudo-Michael reaction of 4,5-dihydro-1*H*-imidazol-2-amines with DEEM was temperature-dependent; therefore, we tried first to perform the corresponding reaction with EMCA at room temperature and then at higher temperatures (up to the boiling point of propan-2-ol). Theoretically, formation of the mixture of isomeric N3 and N6 enamines is possible in the case of both Michael reagents, but in the case of EMCA we observed the formation of N3 enamines neither at −10 °C nor at higher temperatures. The N6 enamines **5a**, **5c**–**5h** formed exclusively underwent cyclization to 1-aryl-5-oxo-2,3-dihydroimidazo[1,2-*a*]pyrimidine-6-carbonitriles **6a**, **6c**–**6h** when the pseudo-Michael reaction was performed at ambient or higher temperature (the derivative **6b** could not be obtained by this method as the preparation of enamine **5b** was not successful). On the contrary, in the case of DEEM, at room temperature the reaction yielded mixtures with varying ratio of isomeric 1-aryl-5-oxo-2,3-dihydroimidazo[1,2-*a*]pyrimidine-6-carboxylates and 1-aryl-7-oxo-2,3-dihydroimidazo[1,2-*a*]pyrimidine-6-carboxylates. Alternatively, to obtain compounds **6a** and **6c**–**6h**, the enamines **5a** and **5c**–**5h** should be heated in boiling acetic acid (Scheme 2). In such conditions the cyclization takes place via the ester group of **5a** and **5c**–**5h**, giving **6a** and **6c**–**6h** exclusively. Myiamoto [41], when performing the similar cyclization reaction under acidic conditions (MeOH saturated with HCl), observed the cyclization via cyano group only, leading to appropriate imines. Analogs of **6a**–**6h** were obtained by Myiamoto [41] in basic conditions (Et_3_N/MeOH).

**Figure:**
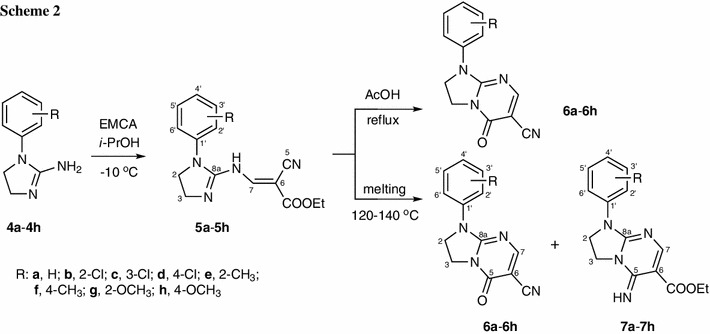

In the next stage we heated enamines **5a** and **5c**–**5h** to 120–140 °C without a solvent. In such conditions the mixtures of the cyclization products both via ester group (**6a** and **6c**–**6h**) and via cyano group, 1-aryl-5-imino-2,3-dihydroimidazo[1,2-*a*]pyrimidine-6-carboxylates (**7a** and **7c**–**7h**) were obtained (Scheme 2). The mixtures were separated with preparative thin-layer chromatography. After preparative TLC separation, compounds **7a** and **7c**–**7h** were extracted from silica gel with methanol. During extraction process some transesterification occurred which resulted in mixtures of ethyl and methyl esters. The percentage of methyl ester in the mixture depended on the substituent in the phenyl group and varied from 10 to 45 % by NMR. The obtained mixtures of ethyl and methyl esters were practically impossible to separate, and thus, they were analysed as received.

Finally, the reactions of respective 4,5-dihydro-1*H*-imidazol-2-amine hydrobromides **3a**–**3h** with EMCA in the presence of base (Et_3_N) led to reaction on N6 nitrogen atom (which is in agreement with our previous results obtained for DEEM [29]) and cyclization via the ester group, resulting in compounds **6a**–**6h** (Scheme 3). This method made it possible to obtain the derivative **6b** with 2-chloro substituent.

**Figure:**
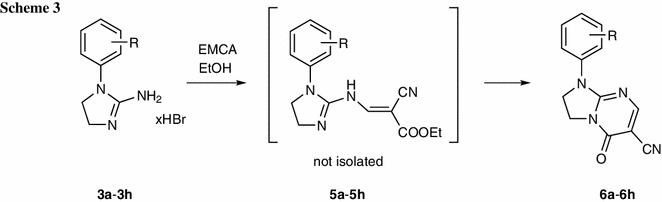

The course of all reactions was confirmed by elementary analysis and spectral data (^1^H and ^13^C NMR, MS). All the compounds were characterized with the aid of NMR spectroscopy. Assignments of ^1^H and ^13^C chemical shifts and ^1^H–^1^H coupling constants were achieved by a combination of several 2D NMR techniques. Since there are not that many protons in heterocyclic rings, the most important experiment for assignments was HMBC spectrum. By HMBC, one can see a long range correlation between H-2 and aromatic substituent at N-1. This proves the assignment of H-2 and H-3 protons. The protons and the carbons at the aromatic substituent are easily assigned by DQF-COSY and HSQC spectra. The most difficult part is the assignment of quaternary carbons at positions 5, 6, and 8a. H-7 showed HMBC correlations to all of those. However, C-8a can be identified by a long range correlation from both H-2 and H-3. A correlation between H-3 and C-5 was not observed, so the assignments of C-5 and C-6 were done based on the very different chemical shift values. Therefore, as an example, the structures of **5f** and **7f** were modeled by DFT method B3LYP/6-31G(d,p) and the NMR chemical shifts were calculated by the same method [42]. The good agreement (the results are not shown here) with experimental values proved the correct assignments. The multiplicities of proton signals H-2 and H-3 are noteworthy. Those methylene protons at positions 2 (H-2a and H-2b) and 3 (H-3a and H-3b) have nearly the same chemical shifts, respectively. However, the vicinal coupling constants between H-2 and H-3 differ. Actually there are four coupling constants: *J*_2a,3a_, *J*_2a,3b_, *J*_2b,3a_, and *J*_2b,3b_ and the signals can be analysed, for example by simulation and iteration software PERCH [43].^1^ It was found that they are all between 7 and 12 Hz. This makes the signals look roughly like triplets, but the unequal coupling constants as well as the second order effects make the signals rather complicated. Therefore, these are stated as multiplets (m) at the experimental section.

The primary fragmentations of compounds **5a** and **5c**–**5h** as well as **7c**–**7h** are mainly initiated from the ester function (see “Experimental”) or via the [M–H]^+^ ions. As mentioned in “Experimental”, compounds **7** were transesterified into mixtures of Me- and Et-esters when eluted with methanol. Therefore, they show also variable amounts of the M^+·^ ions of the methyl esters. On the other hand, with the exception of the [M–H]^+^ ions, the other fragments appear almost always at the same *m/z*-values, e.g. [M–MeOH]^+·^ and [M–EtOH]^+·^ ions. The ion structures were determined by accurate mass measurements and the fragmentation routes with B/E spectra. When comparing the fragmentation patterns of compounds **5** and **7** with each other it appears that compounds **5** may partially rearrange into compounds **7** under EI conditions (see “Experimental”). However, some differences still exist. M^+·^ is the base peak only for **5** **g** but in case of compounds **7** for **7e**, **7** **g**, and **7** **h**. The base peak for **5a**, **5e**, **5f**, and **5** **h** is [M–C_2_H_4_CO_2_]^+·^ but only for **7d** and **7f**. The clearest difference is seen in the abundance of [M–ROH]^+·^ ion (R = Et for **5** and Me or Et for **7**) which is by far more abundant for compounds **5** being even the base peak for **5d** and **5e**. Compounds **5e** and **5** **g** show some ions which are indicative for the 2-Me and 2-OMe substitutions and are missing from the spectra of **5f** and **5** **g**. For **5e** these ions are [M–Me]^+^ at *m/z* 283 (4 %) and [M–Me–EtOH]^+^ at *m/z* 237 (29) and for **5** **g** [M–H–Me]^+·^, [M–Et]^+^, and [M–OMe]^+^ at *m/z* 298 (5), 285 (5), and 283 (36 %), respectively. Similarly 2-Me derivative **7e** gives the ion [M–Me] at *m/z* 269 (8 %) and the Et- and Me-esters the ions [M–Me–EtOH]^+^ and [M–Me–MeOH]^+^ at *m/z* 237 (7 %) although the latter is weaker than that for **5e**. Compound **7g** with 2-OMe substitution gives the ions [M–Me]^+·^ and [M–OMe]^+^ at *m/z* 299 (14 %) and 285 (5 %), 283 (33 %) and 269 (47 %) for Et- and Me-esters, respectively. It should be mentioned that *m/z* 269 corresponds also [M–OEt]^+^ for the Et-ester (**7g**).

Compounds **6a**–**6h** understandably behave differently. M^+·^ is always the base peak except the 2-Cl derivative **6b** where [M–Cl]^+^ ion is the base peak due to the ortho effect. Another ion which originates via the ortho effect is [M–Cl–C_2_H_2_]^+^ at *m/z* 211 (10 %). Similarly 2-Me derivative **6e** shows one ion which is indicative for the ortho methyl, namely [M–Me]^+^ at *m/z* 237 (24 %), which is absent from the mass spectrum of 4-Me derivative **6f**. The presence of ortho-OMe substitution in **6g** is reflected by the ions [M–OMe]^+^ at *m/z* 237 (92 %) and [M–CH_2_O]^+·^ at *m/z* 238 (20 %) which are missing from the spectrum of **6h** except for a small amount of ion 237 (9 %). On the other hand, the 4-OMe derivative **6h** shows the ions [M–Me]^+^ at *m/z* 253 (32 %) and [M–Me–CO]^+^ at *m/*z 225 (7 %) which are missing from the spectrum of **6g**.

**Fig. 1:**
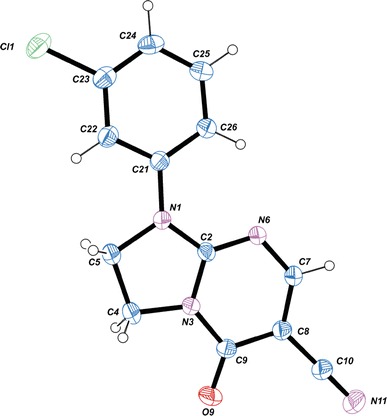
A view of the X-ray molecular structure of **6c** with the atomic labeling scheme

**Fig. 2:**
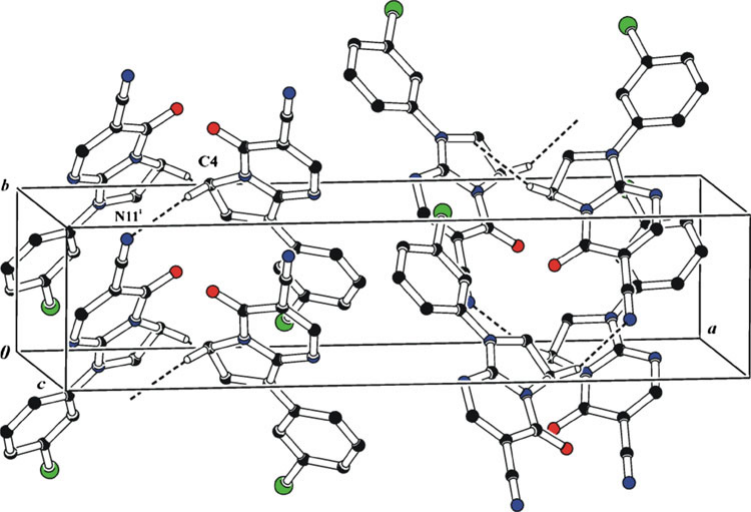
A view of part of the crystal structure of **6c**. *Dashed lines* indicate intermolecular hydrogen bonds

We also succeeded in the preparation of crystals suitable for X-ray structure determination for **6c** and **6d** [44]. The X-ray analyses of compounds **6c** and **6d** were performed in order to confirm the synthetic pathway and the position of the oxo group (5-oxo/7-oxo; Scheme 2). The crystal structure of **6c** is shown in Fig. 1. It was found that the investigated compounds contain an oxo group at position 5. The geometric parameters (bond lengths, angles, torsion angles, and planarity of the rings) are very similar to those observed in a previously reported structure of 1-(4-chlorophenyl)-5-oxo-1,2,3,5-tetrahydroimidazo[1,2-*a*]pyrimidine-6-carbonitrile (**6d**) [44]. The molecule as a whole adopts a nearly planar conformation with the torsion angle C2–N1–C21–C26 of 1.2(4)°. This conformation is stabilized by an intramolecular C26–H26···N6 interaction leading to the formation of a six-member ring described by the S(6) graph-set symbol [2, 45]; C26–H26 = 0.93, H26···N6 = 2.25, C26···N6 = 2.894(4) Å, and C26–H26···N6 = 126°. In the crystal structure, the molecules related by *c* glade plane and *b* translation are linked to form C(7) chains along [011] direction by C4-H4B…N11^(*i*)^ intermolecular hydrogen bonds (C4–H4B = 0.97, H4B···N11 = 2.56, C4···N11 = 3.281(5) Å, C4–H4B···N11 = 132° and (*i*) = ½−*x*, −1 + *y*, −½ + *z*) (Fig. 2). Moreover, the *π*-electron systems of the pairs of pyrimidine ring at (*x*, *y*, *z*) and phenyl ring at (*x*, 1 + *y*, *z*) and phenyl ring at (*x*, *y*, *z*) and pyrimidine ring at (*x*, −1 + *y*, *z*) overlap each other, with centroid-to-centroid separation of 3.5695(18) Ǻ. The *π*···*π* distances between overlapping planes are alternately 3.4648(11) and 3.4564(13) Å and the angle between them is 3.21(14)°.

Theoretical calculations at DFT/B3LYP/6-311 ++G(d,p) ab initio level [42] show that that 5-oxo isomeric form of **6c** and **6d** (the initial geometries were built from their crystallographic data) obtained after energy minimization and geometry optimization in the gaseous phase is more energetically stable than 7-oxo form, with a difference in the energy between the 7-oxo and 5-oxo forms of 45.6 and 46.0 kJ mol^−1^ for **6c** and **6d**, respectively. In solution (water (*ε* = 78.35) and chloroform (*ε* = 4.71), CPCM model [46]) the energy difference between form 7-oxo and 5-oxo is 18.8 kJ mol^−1^ (aqueous solution) and 27.2 kJ mol^−1^ (chloroform solution) for **6c** and 19.3 kJ mol^−1^ (aqueous solution) and 27.6 kJ mol^−1^ (chloroform solution) for **6d**. Thus, the population of the 7-oxo form in vacuum and polar (water) and non-polar (chloroform) solutions estimated using a non-degenerate Boltzmann distribution is below the threshold of the detectability of conventional analytical methods.

## Experimental

All reagents and solvents were purchased and used without additional purification. In particular, EMCA was purchased from Merck. Reactions were routinely monitored by thin-layer chromatography (TLC) in silica gel (60 F_254_ Merck plates, DS horizontal chamber, Chromdes, Lublin, Poland) in toluene-ethyl acetate–methanol (1:3:0.5) eluent system and the products were visualized with ultraviolet light of 254 nm wavelength.

NMR spectra were acquired using Bruker Avance 500 spectrometer (equipped with BBO 5 mm Z-grad probe) operating at 500.13 MHz for ^1^H and 125.77 MHz for ^13^C. Spectra were recorded at 25 °C using DMSO-*d*_*6*_ as solvent with a non-spinning sample in 5 mm NMR tubes. Spectra were processed by a PC with Windows XP operating system and TopSpin software. Proton and carbon spectra were referenced to tetramethylsilane (TMS: 0.00 ppm). In addition to normal ^1^H and ^13^C NMR spectra, also a variety of gradient selected 2D measurements were used to receive an unequivocal assignment of all compounds. DQF-COSY spectra were acquired with cosygpmfqf pulse program (pulse programs refer to original ones installed by Bruker) and NOESY spectra were acquired with noesygpph pulse program with mixing time of 300 ms. ^1^H-^13^C HSQC spectra were acquired with hsqcetgpsisp.2 pulse program (using shaped pulses) with 145 Hz one-bond coupling constant. ^1^H-^13^C HMBC spectra were acquired with hmbcgplpndqf pulse program with 10 Hz long-range coupling constant. Computational methods were used to confirm the assignment of some quaternary carbons. The geometry optimizations and NMR chemical shift calculations were done by density functional B3LYP equipped with basis set 6-31G(d,p). Calculations were done by Gaussian 03 W software [42]. Numbering of atoms for **5a**–**5g** in NMR assignments corresponds to the numbering of the final products **6a**–**6g** and **7a**–**7g** and is shown in Scheme 2.

The electron ionization (EI) mass spectra were recorded on a VG Analytical (Manchester, UK) ZABSpec instrument, equipped with Opus data system. Samples were introduced using a direct insertion probe at ambient temperature. Accurate mass measurements were performed at a resolving power of 8,000–10,000 (10 % valley definition) using peak matching technique and perfluorokerosene (PFK) as a reference compound. The elementary analyses were performed on a Perkin-Elmer analyzer. Melting points were determined with a Boetius apparatus.

X-ray data of **6c** were collected on a Kuma KM4 four-circle diffractometer at room temperature; crystal sizes 0.60 × 0.30 × 0.20 mm, CuK*α* (*λ* = 1.54178 Å) radiation, *ω*–2*θ* scans. The XABS2 absorption correction was applied [47]; *T*_min_ = 0.2071, *T*_max_ = 0.7898. The structure was solved by direct methods using SHELXS97 [48] and refined by full-matrix least-squares with SHELXL97 [48]. The H atoms were positioned geometrically and treated as riding on their parent C atoms with C–H distances of 0.93 Å (aromatic) and 0.97 Å (CH_2_). All H atoms were refined with isotropic displacement parameters taken as 1.5 times those of the respective parent atoms. The flack parameter of −0.02(2) confirmed that the correct absolute structure was refined [49]. All calculations were performed using WINGX version 1.64.05 package [50]. CCDC 912327 contains the supplementary crystallographic data for this paper. These data can be obtained free of charge at http://www.ccdc.cam.ac.uk/conts/retrieving.html (or from the Cambridge Crystallographic Data Centre (CCDC), 12 Union Road, Cambridge CB2 1EZ, UK; fax: +44(0) 1223 336 033; email: deposit@ccdc.cam.ac.uk).

### General procedure to obtain compounds **5a–5h**

1-Aryl-4,5-dihydro-1*H*-imidazol-2-amine (**4a**–**4h**, 0.01 mol) was dissolved in 40 cm^3^ of propan-2-ol and cooled down to the temperature of −10 °C. A solution of 1.69 g of EMCA (**1**, 0.01 mol) in 40 cm^3^ of propan-2-ol was added dropwise at constant stirring. The reaction mixture was stirred at −10 °C for 2 h. The precipitation obtained was filtered off and washed a few times with *n*-hexane, toluene, and a mixture of toluene-ethyl acetate (10:1).

#### *Ethyl 2*-*cyano*-*3*-*[(1*-*phenyl*-*4,5*-*dihydro*-*1H*-*imidazol*-*2*-*yl)amino]prop*-*2*-*enoate* (**5a**, C_15_H_16_N_4_O_2_)

According to general method with 1.61 g of **4a**. The method afforded 1.42 g (60 %) of **5a**. M.p.: 133–134 °C; *R*_*f*_ = 0.48 (toluene/ethyl acetate/methanol 1:3:0.5); ^1^H NMR (500 MHz, DMSO-*d*_6_): *δ* = 8.82 (br s, 1H, H-8), 8.56 (s, 1H, H-7), 7.69 (d, 2H, *J* = 7.7 Hz, H-2′, H-6′), 7.36 (t, 2H, *J* = 7.7 Hz, H-3′, H-5′), 7.14 (t, 1H, *J* = 7.7 Hz, H-4′), 4.14 (m, 2H, H-2), 4.14 (q, 2H, *J* = 7.1 Hz, OC*H*_*2*_CH_3_), 3.69 (m, 2H, H-3), 1.22 (t, 3H, *J* = 7.1 Hz, OCH_2_C*H*_*3*_) ppm; ^13^C NMR (125 MHz, DMSO-*d*_6_): *δ* = 165.37 (C=O), 164.55 (C-7), 161.23 (C-8a), 138.82 (C-1′), 128.38 (C-3′, C-5′), 123.96 (C-4′), 120.86 (C-2′, C-6′), 117.30 (C-5), 82.30 (C-6), 59.56 (O*C*H_2_CH_3_), 47.83 (C-2), 40.31 (C-3), 14.33 (OCH_2_ *C*H_3_) ppm; MS (EI): M^+·^ C_15_H_16_N_4_O_2_^+·^ calc. 284.1273, found 284.1272; *m/z* (%): M^+·^ 284 (65), [M–H]^+^ 283 (7), [M–OEt]^+^ 239 (22), [M–EtOH]^+·^ 238 (26), [M–H–EtOH]^+^ 237 (36), [M–C_2_H_4_OOC]^+·^ 212 (100), [M–EtOOC]^+^ 211 (6), 119 (6), 118 (8), 106 (30), 105 (12), 104 (13), 91 (12), 77 (42).

#### *Ethyl 3*-*[[1*-*(3*-*chlorophenyl)*-*4,5*-*dihydro*-*1H*-*imidazol*-*2*-*yl]amino]*-*2*-*cyanoprop*-*2*-*enoate* (**5c**, C_15_H_15_ClN_4_O_2_)

According to general method with 1.95 g of **4c**. The method afforded 1.52 g (56 %) of **5c**. M.p.: 128–129 °C; *R*_*f*_ = 0.50 (toluene/ethyl acetate/methanol 1:3:0.5); ^1^H NMR (500 MHz, DMSO-*d*_6_): *δ* = 8.96 (br s, 1H, H-8), 8.56 (s, 1H, H-7), 8.01 (t, 1H, *J* = 1.9 Hz, H-2′), 7.60 (dd, 1H, *J* = 1.9 Hz, 8.2 Hz, H-6′), 7.38 (t, 1H, *J* = 8.2 Hz, H-5′),7.18 (dd, 1H, *J* = 1.9 Hz, 8.2 Hz, H-4′), 4.15 (m, 2H, H-2), 4.15 (q, 2H, *J* = 7.0 Hz, OC*H*_*2*_CH_3_), 3.68 (m, 2H, H-3), 1.23 (t, 3H, *J* = 7.0 Hz, OCH_2_C*H*_*3*_) ppm; ^13^C NMR (125 MHz, DMSO-*d*_6_): *δ* = 165.16 (C=O), 164.23 (C-7), 160.87 (C-8a), 140.31 (C-1′), 132.98 (C-3′), 129.90 (C-5′), 123.36 (C-4′), 120.30 (C-2′), 118.60 (C-6′), 117.00 (C-5), 83.43 (C-6), 59.70 (O*C*H_2_CH_3_), 47.49 (C-2), 39.40 (C-3), 14.31 (OCH_2_ *C*H_3_) ppm; MS (EI): M^+·^ C_15_H_15_ClN_4_O_2_^+·^ calc. for ^35^Cl 318.0884, found 318.0882; *m/z* (%): M^+·^^37^Cl: 320 (19), [M–H]^+^ + isotopic 319 (12), [M–OEt]^+^ 275 (6), [M^·^EtOH]^+·^ + isotopic 274 (7), [M–H–EtOH]^+^ 273 (23), [M–C_2_H_4_OOC]^+·^ 248 (32), [M–H–C_2_H_4_OOC]^+·^ + isotopic 247 (21), ^35^Cl: 318 (55), [M–H]^+^ 317 (6), [M–OEt]^+^ 273 (23), [M–EtOH]^+·^ 272 (12), [M–H–EtOH]^+^ 271 (21), [M–C_2_H_4_OOC]^+·^ 246 (100), [M–EtOOC]^+^ 245 (19), 152 (5), 142 (8), 140 (28), 138 (10), 111 (22), 105 (7), 77 (8), 75 (14).

#### *Ethyl 3*-*[[1*-*(4*-*chlorophenyl)*-*4,5*-*dihydro*-*1H*-*imidazol*-*2*-*yl]amino]*-*2*-*cyanoprop*-*2*-*enoate* (**5d**, C_15_H_15_ClN_4_O_2_)

According to general method with 1.95 g of **4d**. The method afforded 1.54 g (57 %) of **5d**. M.p.: 134–136 °C; *R*_*f*_ = 0.48 (toluene/ethyl acetate/methanol 1:3:0.5); ^1^H NMR (500 MHz, DMSO-*d*_6_): *δ* = 8.92 (br s, 1H, H-8), 8.56 (s, 1H, H-7), 7.73 (d, 2H, *J* = 8.6 Hz, H-2′, H-6′), 7.40 (d, 2H, *J* = 8.6 Hz, H-3′, H-5′), 4.14 (m, 2H, H-2), 4.14 (q, 2H, *J* = 7.0 Hz, OC*H*_*2*_CH_3_), 3.68 (m, 2H, H-3), 1.22 (t, 3H, *J* = 7.0 Hz, OCH_2_C*H*_*3*_) ppm; ^13^C NMR (125 MHz, DMSO-*d*_6_): *δ* = 165.26 (C=O), 164.42 (C-7), 160.94 (C-8a), 137.82 (C-1′), 128.16 (C-3′, C-5′), 127.77 (C-4′), 122.33 (C-2′, C-6′), 117.22 (C-5′), 82.85 (C-6), 59.64 (O*C*H_2_CH_3_), 47.69 (C-2), 40.32 (C-3), 14.32 (OCH_2_ *C*H_3_) ppm; MS (EI): M^+·^ C_15_H_15_ClN_4_O_2_^+·^ calc. for ^35^Cl 318.0884, found 318.0885; *m/z* (%): M^+·^^37^Cl: 320 (8), [M–H–EtOH]^+·^ 274 (34), [M–C_2_H_4_OOC]^+·^ 248 (14), [M–H–C_2_H_4_OOC]^+^ + isotopic 247 (9), [M–C_2_H_4_OOC]^+·^ 246 (41), [M–H–C_2_H_4_OOC]^+^ 245 (11), ^35^Cl: 318 (26), ^35^Cl: [M–OEt]^+^ 273 (41), [M–EtOH]^+·^ 272 (100), [M–H–EtOH]^+^ 271 (59), [M–C_2_H_4_OOC]^+·^ 246 (41), [M–EtOOC]^+^ 245 (11), 152 (6), 140 (18), 138 (17), 125 (11), 111 (31), 105 (22), 77 (9), 75 (24).

#### *Ethyl 2*-*cyano*-*3*-*[[1*-*(2*-*methylphenyl)*-*4,5*-*dihydro*-*1H*-*imidazol*-*2*-*yl]amino]prop*-*2*-*enoate* (**5e**, C_16_H_18_N_4_O_2_)

According to general method with 1.75 g of **4e**. The method afforded 1.50 g (60 %) of **5e**. M.p.: 117–118 °C; *R*_*f*_ = 0.49 (toluene/ethyl acetate/methanol 1:3:0.5); ^1^H NMR (500 MHz, DMSO-*d*_6_): *δ* = 8.62 (br s, 1H, H-8), 8.44 (s, 1H, H-7), 7.28 (m, 4H, H-3′, H-4′, H-5′, H-6′), 4.08 (q, 2H, *J* = 7.1 Hz, OC*H*_*2*_CH_3_), 3.98 (m, 2H, H-2), 3.74 (m, 2H, H-3), 2.21 (s, 3H, 2′-CH_3_), 1.18 (t, 3H, *J* = 7.1 Hz, OCH_2_C*H*_*3*_) ppm; ^13^C NMR (125 MHz, DMSO-*d*_6_): *δ* = 165.60 (C=O), 164.82 (C-7), 162.99 (C-8a), 136.89, 135.61 (C-1′, C-2′), 130.75 (C-3′), 127.73, 126.94, 126.53 (C-4′, C-5′, C-6′), 117.22 (C-5), 80.77 (C-6), 59.38 (O*C*H_2_CH_3_), 49.90 (C-2), 40.50 (C-3), 17.62 (2′-CH_3_), 14.32 (OCH_2_ *C*H_3_) ppm; MS (EI): M^+·^ C_16_H_18_N_4_O_2_ calc. 298.1430, found 298.1423; *m/z* (%): M^+·^ 298 (79), [M–H]^+^ 297 (34), [M–OEt]^+^ 253 (31), [M–EtOH]^+·^ 252 (100), [M–H–EtOH]^+^ 251 (99), [M–Me–EtOH]^+^ 237 (29), [M–C_2_H_4_OOC]^+·^ 226 (47), [M–EtOOC]^+^ 225 (22), 223 (8), 158 (82), 157 (18), 156 (10), 132 (11), 131 (15), 130 (16), 120 (13), 118 (31), 117 (22), 116 (13), 91 (55), 77 (21), 65 (40).

#### *Ethyl 2*-*cyano*-*3*-*[[1*-*(4*-*methylphenyl)*-*4,5*-*dihydro*-*1H*-*imidazol*-*2*-*yl]amino]prop*-*2*-*enoate* (**5f**, C_16_H_18_N_4_O_2_)

According to general method with 1.75 g of **4f**. The method afforded 1.62 g (64 %) of **5f**. M.p.: 127–128 °C; *R*_*f*_ = 0.50 (toluene/ethyl acetate/methanol 1:3:0.5); ^1^H NMR (500 MHz, DMSO-*d*_6_): *δ* = 8.77 (br s, 1H, H-8), 8.54 (s, 1H, H-7), 7.55 (d, 2H, *J* = 8.1 Hz, H-2′, H-6′), 7.17 (d, 2H, *J* = 8.1 Hz, H-3′, H-5′), 4.13 (m, 2H, H-2), 4.13 (q, 2H, *J* = 7.0 Hz, OC*H*_*2*_CH_3_), 3.67 (t, 2H, *J* = 8.8 Hz, H-3), 2.29 (s, 3H, 4′-CH_3_), 1.22 (t, 3H, *J* = 7.0 Hz, OCH_2_ *CH*_*3*_) ppm; ^13^C NMR (125 MHz, DMSO-*d*_6_): *δ* = 165.44 (C=O), 164.61 (C-7), 161.26 (C-8a), 136.26 (C-1′), 133.33 (C-4′), 128.83 (C-3′, C-5′), 121.05 (C-2′, C-6′), 117.35 (C-5), 82.04 (C-6), 59.53 (O*C*H_2_CH_3_), 47.99 (C-2), 39.46 (C-3), 20.30 (4′-CH_3_), 14.34 (OCH_2_ *C*H_3_) ppm; MS (EI): M^+·^ C_16_H_18_N_4_O_2_^+·^ calc. 298.1430, found 298.1426; *m/z* (%): M^+·^ 298 (69), [M–H]^+^ 297 (11), [M–OEt]^+^ 253 (23), [M–EtOH]^+·^ 252 (42), [M–H–EtOH]^+^ 251 (41), [M–C_2_H_4_OOC]^+·^ 226 (100), [M–EtOOC]^+^ 225 (25), 224 (7), 120 (35), 132 (6), 131 (6), 118 (13), 117 (7), 105 (14), 91 (42), 77 (10), 65 (20).

#### *Ethyl 2*-*cyano*-*3*-*[[1*-*(2*-*methoxyphenyl)*-*4,5*-*dihydro*-*1H*-*imidazol*-*2*-*yl]amino]prop*-*2*-*enoate* (**5g**, C_16_H_18_N_4_O_3_)

According to general method with 1.91 g of **4** **g**. The method afforded 1.79 g (67 %) of **5** **g**. M.p.: 135–136 °C; *R*_*f*_ = 0.49 (toluene/ethyl acetate/methanol 1:3:0.5); ^1^H NMR (500 MHz, DMSO-*d*_6_): *δ* = 8.60 (br s, 1H, H-8), 8.45 (s, 1H, H-7), 7.32 (dt, 1H, *J* = 1.2 Hz, 7.5 Hz, H-4′), 7.28 (dd, 1H, *J* = 1.2 Hz, 7.8 Hz, H-6′), 6.98 (t, 1H, *J* = 7.4 Hz, H-5′), 7.13 (d, 1H, *J* = 8.2 Hz, H-3′), 4.09 (q, 2H, *J* = 7.0 Hz, OC*H*_*2*_CH_3_), 3.95 (dd, 2H, *J* = 8.5 Hz, 9.6 Hz, H-2), 3.81 (s, 3H, 2′-OCH_3_), 3.71 (dd, 2H, *J* = 8.5 Hz, 9.6 Hz, H-3), 1.18 (t, 3H, *J* = 7.0 Hz, OCH_2_ *CH*_*3*_) ppm; ^13^C NMR (125 MHz, DMSO-*d*_6_): *δ* = 165.71 (C=O), 164.88 (C-7), 163.73 (C-8a), 154.61 (C-2′), 128.82 (C-4′, C-6′), 126.46 (C-1′), 120.38 (C-5′), 117.39 (C-5), 112.59 (C-3′), 80.37 (C-6), 59.34 (O*C*H_2_CH_3_), 55.69 (2′-OCH_3_), 49.14 (C-2), 40.43 (C-3), 14.34 (OCH_2_ *C*H_3_) ppm; MS (EI): M^+·^ C_16_H_18_N_4_O_3_^+·^ calc. 314.1379, found 314.1385; *m/z* (%): M^+·^ 314 (100), [M–H]^+^ 313 (18), [M–H–CH_3_]^+·^ 298 (5), [M–Et]^+^ 285 (5), [M–OCH_3_]^+^ 283 (36), [M–OEt]^+^ 269 (26), [M–EtOH]^+·^ 268 (32), [M–H–EtOH]^+^ 267 (20), [M–C_2_H_4_OOC]^+·^ 242 (79), [M–EtOOC]^+^ 241 (24), 237 (48), 212 (17), 211 (26), 174 (29), 136 (20), 134 (14), 121 (16), 129 (30), 119 (10), 106 (16), 105 (18), 104 (10), 93 (11), 92 (21), 91 (18), 77 (32), 65 (28).

#### *Ethyl 2*-*cyano*-*3*-*[[1*-*(4*-*methoxyphenyl)*-*4,5*-*dihydro*-*1H*-*imidazol*-*2*-*yl]amino]prop*-*2*-*enoate* (**5h**, C_16_H_18_N_4_O_3_)

According to general method with 1.91 g of **4h**. The method afforded 1.55 g (58 %) of **5h**. M.p.: 173–174 °C; *R*_*f*_ = 0.51 (toluene/ethyl acetate/methanol 1:3:0.5); ^1^H NMR (500 MHz, DMSO-*d*_6_): *δ* = 8.66 (br s, 1H, H-8), 8.53 (s, 1H, H-7), 7.55 (d, 2H, *J* = 8.5 Hz, H-2′, H-6′), 6.93 (d, 2H, *J* = 8.5 Hz, H-3′, H-5′), 4.12 (m, 2H, H-2), 4.12 (q, 2H, *J* = 6.7 Hz, OC*H*_*2*_CH_3_), 3.75 (s, 3H, 4′-OCH_3_), 3.67 (t, 2H, *J* = 8.4 Hz, H-3), 1.21 (t, 3H, *J* = 6.7 Hz, OCH_2_ *CH*_*3*_) ppm; ^13^C NMR (125 MHz, DMSO-*d*_6_): *δ* = 165.56 (C=O), 164.71 (C-7), 161.45 (C-8a), 156.10 (C-4′), 131.72 (C-1′), 123.03 (C-2′, C-6′), 117.38 (C-5), 113.62 (C-3′, C-5′), 81.66 (C-6), 59.49 (O*C*H_2_CH_3_), 55.18 (4′-OCH_3_), 48.44 (C-2), 39.40 (C-3), 14.34 (OCH_2_ *C*H_3_) ppm; MS (EI): M^+·^ C_16_H_18_N_4_O_3_^+·^ calc. 314.1379, found 314.1382; *m/z* (%): M^+·^ 314 (87), [M–H]^+^ 313 (22), [M–OEt]^+^ 269 (23), [M–EtOH]^+·^ 268 (32), [M–H–EtOH]^+^ 267 (29), [M–Me–HOEt]^+^ 253 (15), [M–C_2_H_4_OOC]^+·^ 242 (100), [M–EtOOC]^+^ 241 (27), 225 (7), 136 (45), 135 (11), 134 (218), 133 (12), 121 (17), 120 (26), 106 (7), 105 (13), 93 (6), 92 (19), 91 (6), 77 (23), 65 (16).

### General procedure to obtain compounds **6a–6h**

#### Method A

Enamine **5a**–**5** **h** (0.01 mol) was dissolved in 10 cm^3^ of glacial acetic acid and refluxed under mild boiling for 4 h. The solvent was distilled off and the solid residue was crystallized from DMF.

#### Method B

1-Aryl-4,5-dihydro-1*H*-imidazol-2-amine hydrobromides (**3a**–**3** **h**, 0.01 mol) and 1.69 g of EMCA (**1**, 0.01 mol) were dissolved in 20 cm^3^ of ethanol. The solution was stirred under reflux for 4 h and then 1.02 g of triethylamine (0.01 mol) was added dropwise over a period of 15 min and the mixture was refluxed for additional 6 h. The precipitate was filtered off and washed with methanol.

##### *5*-*Oxo*-*1*-*phenyl*-*2,3*-*dihydroimidazo[1,2*-*a]pyrimidine*-*6*-*carbonitrile* (**6a**, C_13_H_10_N_4_O)

According to general method A with 2.36 g of **5a**. The method afforded 1.36 g (57 %) of **6a**. M.p.: 210–213 °C; *R*_*f*_ = 0.62 (toluene/ethyl acetate/methanol 1:3:0.5); ^1^H NMR (500 MHz, DMSO-*d*_6_): *δ* = 8.40 (s, 1H, H-7), 7.72 (d, 2H, *J* = 8.5 Hz, H-2′, H-6′), 7.45 (dd, 2H, *J* = 7.5 Hz, 8.5 Hz, H-3′, H-5′), 7.23 (t, 1H, *J* = 7.5 Hz, H-4), 4.26 (m, 2H, H-2), 4.15 (m, 2H, H-3) ppm; ^13^C NMR (125 MHz, DMSO-*d*_6_): *δ* = 163.57 (C-7), 157.95 (C-5), 155.79 (C-8a), 137.65 (C-1′), 128.83 (C-3′, C-5′), 125.02 (C-4′), 120.38 (C-2′, C-6′), 115.78 (6-CN), 90.55 (C-6), 46.00 (C-2), 40.31 (C-3) ppm; MS (EI): M^+·^ C_13_H_10_N_4_O^+·^ calc. 238.0842, found 238.0855; *m/z* (%): M^+·^ 238 (100), [M–H]^+^ 237 (71), [M–CN]^+^ 212 (7), [M–HCN]^+·^ 211 (4), [M–H–CO]^+^ 209 (5), 105 (10), 104 (5), 91 (7), 77 (17), 54 (9), 51 (8).

##### *1*-*(2*-*Chlorophenyl)*-*5*-*oxo*-*2,3*-*dihydroimidazo[1,2*-*a]pyrimidine*-*6*-*carbonitrile* (**6b**, C_13_H_9_ClN_4_O)

According to general method B with 2.72 g of **5b**. The method afforded 1.64 g (60 %) of **6b**. M.p.: 236-240 °C; *R*_*f*_ = 0.60 (toluene/ethyl acetate/methanol 1:3:0.5); ^1^H NMR (500 MHz, DMSO-*d*_6_): *δ* = 8.26 (s, 1H, H-7),7.65 (m, 2H, H-3′, H-6′), 7.49 (m, 2H, H-4′, H-5′), 4.28 (m, 2H, H-2), 4.14 (m, 2H, H-3) ppm; ^13^C NMR (125 MHz, DMSO-*d*_6_): *δ* = 164.03 (C-7), 157.99 (C-5), 157.54 (C-8a), 134.07 (C-1′), 131.47 (C-2′), 130.43 (C-5′), 130.19 (C-6′), 130.00 (C-3′), 128.40 (C-4′), 115.76 (6-CN), 90.18 (C-6), 47.63 (C-2), 41.45 (C-3) ppm; MS (EI): M^+·^ C_13_H_9_ClN_4_O^+·^ calc. for ^35^Cl 272.0465, found 272.0477; *m/z* (%): M^+·^^37^Cl: 274 (8), ^35^Cl: 272 (26), [M–Cl]^+^ 237 (100), [M–Cl–C_2_H_2_]^+^ 211 (10), 138 (3), 111 (6), 105 (4), 75 (5), 54 (7).

##### *1*-*(3*-*Chlorophenyl)*-*5*-*oxo*-*2,3*-*dihydroimidazo[1,2*-*a]pyrimidine*-*6*-*carbonitrile* (**6c**, C_13_H_9_ClN_4_O)

According to general method A with 2.72 g of **5c**. The method afforded 1.47 g (54 %) of **6c**. M.p.: 245–248 °C; *R*_*f*_ = 0.65 (toluene/ethyl acetate/methanol 1:3:0.5); ^1^H NMR (500 MHz, DMSO-*d*_6_): *δ* = 8.46 (s, 1H, H-7), 7.98 (t, 1H, *J* = 2.0 Hz, H-2′), 7.64 (dd, 1H, *J* = 2.0 Hz, 8.1 Hz, H-6′), 7.48 (t, 1H, *J* = 8.1 Hz, H-5′), 7.29 (dd, 1H, *J* = 2.0 Hz, 8.1 Hz, H-4′), 4.26 (m, 2H, H-2), 4.15 (m, 2H, H-3) ppm; ^13^C NMR (125 MHz, DMSO-*d*_6_): *δ* = 163.42 (C-7), 157.79 (C-5), 155.68 (C-8a), 139.15 (C-1′), 133.20 (C-3′), 130.50 (C-5′), 124.49 (C-4′), 119.72 (C-2′), 118.35 (C-6′), 115.57 (6-CN), 91.27 (C-6), 45.84 (C-2), 40.32 (C-3) ppm; MS (EI): M^+·^ C_13_H_9_ClN_4_O^+·^ calc. for ^35^Cl 272.0465, found 272.0467; *m/z* (%): M^+·^^37^Cl: 274 (32), ^35^Cl: 272 (100), [M–H]^+^^37^Cl: 273 (35), ^35^Cl: 271 (60), [M–H–CO]^+^ 243 (4), [M–HCl]^+·^ 246 (12), 138 (5), 125 (5), 111 (11), 105 (10), 75 (8), 54 (11).

Crystal data: C_13_H_9_ClN_4_O, *M* = 272.69, orthorhombic, space group *Pca*2_1_, *a* = 22.8305(14) Å, *b* = 5.4318(8) Å, *c* = 9.5141(6) Å, *V* = 1,179.9(2) Å^3^, *Z* = 4, *d*_calc_ = 1.535 Mg m^−3^, *F*(000) = 560, *μ*(Cu K*α*) = 2.851 mm^−1^, *T* = 293 K, 1825 measured reflections (*θ* range 3.87–80.17°), 1,433 unique reflections (*R*_int_ = 0.051), final *R* = 0.033, *wR* = 0.099, *S* = 1.091 for 1304 reflections with *I* > 2*σ*(*I*).

##### *1*-*(4*-*Chlorophenyl)*-*5*-*oxo*-*2,3*-*dihydroimidazo[1,2*-*a]pyrimidine*-*6*-*carbonitrile* (**6d**, C_13_H_9_ClN_4_O)

According to general method A with 2.72 g of **5d**. The method afforded 2.05 g (75 %) of **6d**. M.p.: 214–216 °C; *R*_*f*_ = 0.62 (toluene/ethyl acetate/methanol 1:3:0.5); ^1^H NMR (500 MHz, DMSO-*d*_6_): *δ* = 8.42 (s, 1H, H-7), 7.77 (d, 2H, *J* = 9.0 Hz, H-2′, H-6′), 7.51 (d, 2H, *J* = 9.0 Hz, H-3′, H-5′), 4.24 (m, 2H, H-2), 4.14 (m, 2H, H-3) ppm; ^13^C NMR (125 MHz, DMSO-*d*_6_): *δ* = 163.41 (C-7), 157.83 (C-5), 155.63 (C-8a), 136.65 (C-1′), 128.72 (C-3′, C-4′), 121.70 (C-2′, C-6′), 115.64 (6-CN), 91.00 (C-6), 45.89 (C-2), 40.30 (C-3) ppm; MS (EI): M^+·^ C_13_H_9_ClN_4_O^+·^ calc. for ^35^Cl 272.0465, found 272.0463; *m/z* (%): M^+·^^37^Cl: 274 (33), ^35^Cl: 272 (100), [M–H]^+^^37^Cl: 273 (29), ^35^Cl: 271 (48), [M–H–CO]^+^ 243 (3), C_7_H_5_ClN^+^ 138 (6), C_7_H_6_Cl^+^ 125 (5), C_6_H_4_ClN^+^ 111 (10), C_5_H_3_N_3_^+^ 105 (10), C_6_H_3_^+^ 75 (7), C_2_H_2_N_2_^+^ 54 (10).

##### *1*-*(2*-*Methylphenyl)*-*5*-*oxo*-*2,3*-*dihydroimidazo[1,2*-*a]pyrimidine*-*6*-*carbonitrile* (**6e**, C_14_H_12_N_4_O)

According to general method A with 2.51 g of **5e**. The method afforded 1.94 g (77 %) of **6e**. M.p.: 204–206 °C; *R*_*f*_ = 0.57 (toluene/ethyl acetate/methanol 1:3:0.5); ^1^H NMR (500 MHz, DMSO-*d*_6_): *δ* = 8.21 (s, 1H, H-7), 7.40 (dd, 1H, *J* = 1.8 Hz, 7.2 Hz, H-6′), 7.30–7.35 (m, 3H, H-3′, H-4′, H-5′), 4.23 (m, 2H, H-3), 4.10 (m, 2H, H-2), 2.23 (s, 3H, CH_3_) ppm; ^13^C NMR (125 MHz, DMSO-*d*_6_): *δ* = 164.10 (C-7), 158.22 (C-5), 157.21 (C-8a), 135.88 (C-2′), 135.48 (C-1′), 130.89 (C-3′), 128.59 (C-4′), 127.20 (C-6′), 126.88 (C-5′), 116.04 (6-CN), 89.31 (C-6), 48.01 (C-2), 41.28 (C-3), 17.23 (2′-CH_3_) ppm; MS (EI): M^+·^ C_14_H_12_N_4_O^+·^ calc. 252.1001, found 252.1011; *m/z* (%): M^+·^ 252 (100), [M–H]^+^ 251 (88), [M–CH_3_]^+^ 237 (24), [M–C_2_H_5_]^+^+[M–H–CO]^+^ 223 (4), C_10_H_10_N_2_^+^ 158 (45), C_9_H_9_N^+^ 131 (5), 130 (5), C_8_H_8_N^+^ 118 (6), 117 (6), 91 (13), C_5_H_5_^+·^ 65 (9), 54 (6).

##### *1*-*(4*-*Methylphenyl)*-*5*-*oxo*-*2,3*-*dihydroimidazo[1,2*-*a]pyrimidine*-*6*-*carbonitrile* (**6f**, C_14_H_12_N_4_O)

According to general method A with 2.51 g of **5f**. The method afforded 1.51 g (63 %) of **6f**. M.p.: 250–254 °C; *R*_*f*_ = 0.62 (toluene/ethyl acetate/methanol 1:3:0.5); ^1^H NMR (500 MHz, DMSO-*d*_6_): *δ* = 8.38 (s, 1H, H-7), 7.59 (d, 2H, *J* = 8.5 Hz, H-2′, H-6′), 7.25 (d, 2H, *J* = 8.5 Hz, H-3′, H-5′), 4.23 (m, 2H, H-2), 4.14 (m, 2H, H-3), 2.31 (s, 3H, CH_3_) ppm; ^13^C NMR (125 MHz, DMSO-*d*_6_): *δ* = 163.62 (C-7), 157.98 (C-5), 155.77 (C-8a), 135.14 (C-1′), 134.42 (C-4′), 129.24 (C-3′, C-5′), 120.48 (C-2′, C-6′), 115.85 (6-CN), 90.22 (C-6), 46.13 (C-2), 40.29 (C-3), 20.34 (4′-CH_3_) ppm; MS (EI): M^+·^ C_14_H_12_N_4_O^+·^ calc. 252.1011, found 252.1019; *m/z* (%): M^+·^ 252 (100), [M–H]^+^ 251 (53), [M–H–CO]^+^ 223 (4), 118 (4), 105 (9), 91 (12), 65 (4), 54 (6).

##### *1*-*(2*-*Methoxyphenyl)*-*5*-*oxo*-*2,3*-*dihydroimidazo[1,2*-*a]pyrimidine*-*6*-*carbonitrile* (**6g**, C_14_H_12_N_4_O_2_)

According to general method A with 2.67 g of **5** **g**. The method afforded 1.61 g (61 %) of **6** **g**. M.p.: 240–243 °C; *R*_*f*_ = 0.55 (toluene/ethyl acetate/methanol 1:3:0.5); ^1^H NMR (500 MHz, DMSO-*d*_6_): *δ* = 8.22 (s, 1H, H-7), 7.41 (d, 1H, *J* = 7.5 Hz, H-6′), 7.40 (dt, 1H, *J* = 1.4 Hz, 7.5 Hz, H-4′), 7.18 (d, 1H, *J* = 7.5 Hz, H-3′), 7.03 (dt, 1H, *J* = 1.4 Hz, 7.5 Hz, H-5′), 4.22 (m, 2H, H-3), 4.08 (m, 2H, H-2), 3.82 (s, 3H, OCH_3_) ppm; ^13^C NMR (125 MHz, DMSO-*d*_6_): *δ* = 164.12 (C-7), 158.14 (C-5), 157.67 (C-8a), 154.72 (C-2′), 129.74 (C-4′), 128.62 (C-6′), 124.78 (C-1′), 120.53 (C-5′), 116.01 (6-CN), 112.56 (C-3′), 89.43 (C-6), 55.71 (2′-OCH_3_), 47.30 (C-2), 41.16 (C-3) ppm; MS (EI): M^+·^ C_14_H_12_N_4_O_2_^+·^ calc. 268.0960, found 268.0960; *m/z* (%): M^+·^ 268 (100), [M–H]^+^ 267 (22), [M–H–CH_3_]^+^ 252 (6), [M–H–H_2_O]^+^ 249 (10), [M–H–CO]^+^ 239 (13), C_10_H_10_N_2_O^+^ 174 (10), C_8_H_8_NO^+^ 134 (5), C_6_H_6_N^+^+C_6_H_4_O^+^ (2:5) 92 (5), C_6_H_5_N^+^+ C_7_H_7_^+^ (1:3) 91 (5), 78 (6), 77 (9), 65 (7), 54 (8), 52 (5), 51 (6).

##### *1*-*(4*-*Methoxyphenyl)*-*5*-*oxo*-*2,3*-*tetrahydroimidazo[1,2*-*a]pyrimidine*-*6*-*carbonitrile* (**6h**, C_14_H_12_N_4_O_2_)

According to general method A with 2.67 g of **5** **h**. The method afforded 1.45 g (54 %) of **6** **h**. M.p.: 218–220 °C; *R*_*f*_ = 0.57 (toluene/ethyl acetate/methanol 1:3:0.5); ^1^H NMR (500 MHz, DMSO-*d*_6_): *δ* = 8.33 (s, 1H, H-7), 7.58 (d, 2H, *J* = 8.9 Hz, H-2′, H-6′), 7.00 (d, 2H, *J* = 8.9 Hz, H-3′, H-5′), 4.20 (m, 2H, H-2), 4.13 (m, 2H, H-3), 3.76 (s, 3H, OCH_3_) ppm; ^13^C NMR (125 MHz, DMSO-*d*_6_): *δ* = 163.72 (C-7), 158.04 (C-5), 156.83 (C-4′), 155.91 (C-8a), 130.44 (C-1′), 122.77 (C-2′, C-6′), 115.93 (6-CN), 114.04 (C-5′), 114.04 (C-3′), 89.85 (C-6), 55.26 (4′-OCH_3_), 46.62 (C-2), 40.34 (C-3) ppm; MS (EI): M^+·^ C_14_H_12_N_4_O_2_^+·^ calc. 268.0960, found 268.0969; *m/z* (%): M^+·^ 268 (100), [M–H]^+^ 267 (14), [M–CH_3_]^+^ 253 (32), [M–H–CH_3_]^+^ 252 (6), [M–CH_3_O]^+^ 237 (9), [M–CH_3_–CO]^+^ 225 (7), 134 (9), 105 (4), 77 (5), 54 (6).

### General procedure to obtain compounds **7a** and **7c**–**7h**

Compound **5a**–**5** **h** (0.01 mol) was carefully heated in a porcelain evaporating dish to complete melting and subsequent solidification. After the chromatographic control of the completeness of melting the mixture was dissolved in 4 cm^3^ of DMF and applied on two preparative 20 × 20 TLC plates (Merck). Each plate was developed twice with toluene-ethyl acetate–methanol (1:3:0.5) eluent system and then four times with a stronger eluent system containing higher amount of the methanol (1:3:1) until the complete separation of two products of melting (visualization in the UV light at the wavelength of 254 nm). The band containing the adsorbed imines (mixtures of ethyl and methyl esters) was removed with the silica support and was three times extracted with boiling ethanol for 30 min and then the solvent was evaporated. Thus, compounds **7a** and **7c**–**7** **h** were obtained as mixtures of ethyl and methyl esters.

#### *Ethyl (methyl) 5*-*imino*-*1*-*phenyl*-*2,3*-*dihydroimidazo[1,2*-*a]pyrimidine*-*6*-*carboxylate* (**7a**, C_15_H_16_N_4_O_2_)

According to general method with 2.36 g of **5a**. The method afforded 0.33 g (23 %) of **7a** (a 90:10 mixture of the Et- and Me-esters based on the intensity of the CH_2_-signals in the NMR spectrum. The abundance ratio of the M^+·^ ions 86:14). M.p.: 130–132 °C; *R*_*f*_ = 0.00 (toluene/ethyl acetate/methanol 1:3:0.5); ^1^H NMR (500 MHz, DMSO-*d*_6_): *δ* = 8.17 (s, 1H, H-7), 8.11 (br s, 1H, 5-N*H*), 7.74 (d, 2H, *J* = 7.4 Hz, H-2′, H-6′), 7.42 (t, 2H, *J* = 7.4 Hz, H-3′, H-5′), 7.18 (t, 1H, *J* = 7.4 Hz, H-4′), 4.23 (m, 2H, H-2), 4.20 (q, 2H, *J* = 7.1 Hz, OC*H*_*2*_CH_3_), 4.03 (m, 2H, H-3), 1.26 (t, 3H, *J* = 7.1 Hz, OCH_2_C*H*_*3*_) ppm; ^13^C NMR (125 MHz, DMSO-*d*_6_): *δ* = 165.23 (C=O), 157.40 (C-5), 155.27 (C-8a), 152.68 (C-7), 138.35 (C-1′), 128.68 (C-3′, C-5′), 124.16 (C-4′), 119.90 (C-2′, C-6′), 102.66 (C-6), 59.60 (O*C*H_2_CH_3_), 45.68 (C-2), 40.57 (C-3), 14.06 (OCH_2_ *C*H_3_) ppm; MS (EI): calc. for C_15_H_16_N_4_O_2_ 284.1273, found 284.1269; *m/z* (%): Et-ester: M^+·^ 284 (89), [M–H]^+^ 283 (8); Me-ester: M^+·^ 270 (14), [M–H]^+^ 269 (3); [M–EtO]^+^ and [M–MeO]^+^ 239 (22), [M–HOEt]^+^ and [M–HOMe]^+^ 238 (10), [M–H–HOEt]^+·^ and [M–H–HOMe]^+·^ 237 (251), [M–C_2_H_4_OOC]^+·^ and [M–H_2_COOC]^+·^ 212 (100), [M–H–C_2_H_4_OOC]^+^ and [M–H–H_2_COOC]^+^ 211 (15), 161 (12), 160 (11), 149 (7), 118 (5), 106 (34), 105 (50), 104 (7), 91 (7), 77 (23).

#### *Ethyl (methyl) 1*-*(3*-*chlorophenyl)*-*5*-*imino*-*2,3*-*dihydroimidazo[1,2*-*a]pyrimidine*-*6*-*carboxylate* (**7c**, C_15_H_15_ClN_4_O_2_)

According to general method with 2.72 g of **5c**. The method afforded 0.49 g (31 %) of **7c** (a 56:44 mixture of the Et- and Me-esters based on the intensity of the CH_2_-signals in the NMR spectrum. The abundance ratio of the M^+·^ ions was 47:56). M.p.: 153–155 °C; *R*_*f*_ = 0.00 (toluene/ethyl acetate/methanol 1:3:0.5); NMR data for **7c**: ^1^H NMR (500 MHz, DMSO-*d*_6_): *δ* = 8.23 (s, 1H, H-7), 8.18 (br s, 1H, 5-N*H*), 8.00 (m, 1H, H-2′), 7.64 (m, 1H, H-6′), 7.44 (t, 1H, *J* = 8.2 Hz, H-5′), 7.23 (m, 1H, H-4′), 4.22 (m, 2H, H-2), 4.22 (q, 2H, *J* = 7.1 Hz, OC*H*_*2*_CH_3_), 4.03 (m, 2H, H-3), 1.27 (t, 3H, *J* = 7.1 Hz, OCH_2_C*H*_*3*_) ppm; ^13^C NMR (125 MHz, DMSO-*d*_6_): *δ* = 165.11 (C=O), 157.27 (C-7), 155.15 (C-8a), 152.57 (C-5), 139.81 (C-1′), 133.10 (C-3′), 130.36 (C-5′), 123.70 (C-4′), 119.34 (C-2′), 117.90 (C-6′), 102.96 (C-6), 59.76 (O*C*H_2_CH_3_), 45.60 (C-2), 40.69 (C-3), 14.04 (OCH_2_ *C*H_3_) ppm; MS (EI): M^+·^ C_15_H_15_ClN_4_O_2_^+·^ calc. for ^35^Cl (Et-ester) 318.0884, found 318.0885; calc. for ^35^Cl (Me-ester) 304.0727, found 304.0730; *m/z* (%): Et-ester: M^+·^^37^Cl 320 (12), ^35^Cl 318 (37); Me-ester: ^37^Cl 306 (14), ^35^Cl 304 (42), [M–H]^+^ 303 (10); ^37^Cl: [M–OEt]^+^ and [M–OMe]^+^ 275 (8), ^35^Cl: [M–OEt]^+^ and [M–OMe]^+^ 273 (32), [M–HOEt]^+·^ and [M–HOMe]^+·^ 272 (14), [M–H–HOEt]^+^ and [M–H–HOMe]^+^ 271 (29), ^37^Cl: [M–C_2_H_4_OOC]^+·^ and [M–H_2_COOC]^+·^ 248 (31), [M–H–C_2_H_4_OOC]^+·^ and [M–H–H_2_COOC]^+·^ (+isotopic) 247 (21), ^35^Cl: [M–C_2_H_4_OOC]^+·^ and [M–H_2_COOC]^+·^ 246 (100), [M–H–C_2_H_4_OOC]^+·^ and [M–H–H_2_COOC]^+·^ 245 (18), 142 (10), 140 (34), 138 (12), 111 (27).

#### *Ethyl (methyl) 1*-*(4*-*chlorophenyl)*-*5*-*imino*-*2,3*-*dihydroimidazo[1,2*-*a]pyrimidine*-*6*-*carboxylate* (**7d**, C_15_H_15_ClN_4_O_2_)

According to general method with 2.72 g of **5d**. The method afforded 0.43 g (27 %) of **7d** (a 75:25 mixture of Et and Me esters based on the intensity of the CH_2_-signals in the NMR spectrum. The abundance ratio of the M^+·^ ions was 76:24). M.p.: 170–171 °C; *R*_*f*_ = 0.00 (toluene/ethyl acetate/methanol 1:3:0.5); NMR data for **7d**: ^1^H NMR (500 MHz, DMSO-*d*_6_): *δ* = 8.17 (s, 1H, H-7), 8.13 (br s, 1H, 5-N*H*), 7.79 (m, 2H, H-2′, H-6′), 7.48 (m, 2H, H-3′, H-5′), 4.21 (m, 2H, H-2), 4.21 (q, 2H, *J* = 7.1 Hz, OC*H*_*2*_CH_3_), 4.03 (m, 2H, H-3), 1.26 (t, 3H, *J* = 7.1 Hz, OCH_2_C*H*_3_) ppm; ^13^C NMR (125 MHz, DMSO-*d*_6_): *δ* = 165.19 (C=O), 157.13 (C-7), 155.19 (C-8a), 152.58 (C-5), 137.38 (C-1′), 128.56 (C-3′, C-5′), 127.91 (C-4′), 121.28 (C-2′, C-6′), 103.03 (C-6), 59.67 (O*C*H_2_CH_3_), 45.63 (C-2), 40.58 (C-3), 14.06 (OCH_2_ *C*H_3_) ppm; MS (EI): M^+·^ C_15_H_15_ClN_4_O_2_^+·^ calc. for ^35^Cl (Et-ester) 318.0884, found 318.0885, calc. for ^35^Cl (Me-ester) 304.0727, found 304.0732; *m/z* (%): Et-ester: M^+·^^37^Cl 320 (17), ^35^Cl 318 (52), Me-ester: ^37^Cl 306 (5), ^35^Cl 304 (16), [M–H]^+^ 303 (4), ^37^Cl: [M–OEt]^+^ and [M–OMe]^+^ 275 (7), ^35^Cl: [M–OEt]^+^ and [M–OMe]^+^ 273 (28), [M–HOEt]^+·^ and [M–HOMe]^+·^ 272 (12), [M–H–HOEt]^+^ and [M–H–HOMe]^+^ 271 (22), ^37^Cl: [M–C_2_H_4_OOC]^+·^ and [M–H_2_COOC]^+·^ 248 (32), [M–H–C_2_H_4_OOC]^+·^ and [M–H–H_2_COOC]^+·^ (+isotopic) 247 (21), ^35^Cl: [M–C_2_H_4_OOC]^+·^ and [M–H_2_COOC]^+·^ 246 (100), [M–H–C_2_H_4_OOC]^+·^ and [M–H–H_2_COOC]^+·^ 245 (21), 142 (7), 140 (24), 138 (7), 111 (10).

#### *Ethyl (methyl) 5*-*imino*-*1*-*(2*-*methylphenyl)*-*2,3*-*dihydroimidazo[1,2*-*a]pyrimidine*-*6*-*carboxylate* (**7e**, C_16_H_18_N_4_O_2_)

According to general method with 2.51 g of **5e**. The method afforded 0.31 g (21 %) of **7e** (a 70:30 mixture of the Et- and Me-esters based on the intensity of the CH_2_-signals in the NMR spectrum and a 72:28 mixture 86:14 mixture based on the abundance of the M^+·^ ions). M.p.: 155–160 °C; *R*_*f*_ = 0.00 (toluene/ethyl acetate/methanol 1:3:0.5); NMR data for **7e**: ^1^H NMR (500 MHz, DMSO-*d*_6_): *δ* = 8.09 (br s, 1H, 5-N*H*), 8.02 (s, 1H, H-7), 7.40 − 7.26 (m, 4H, H-3′, H-4′, H-5′, H-6′), 4.17 (q, 2H, *J* = 7.1 Hz, OC*H*_*2*_CH_3_), 4.11 (m, 2H, H-2), 4.08 (m, 2H, H-3), 2.22 (s, 3H, 2′-CH_3_), 1.23 (t, 3H, *J* = 7.1 Hz, OCH_2_C*H*_3_) ppm; ^13^C NMR (125 MHz, DMSO-*d*_6_): *δ* = 165.42 (C=O), 158.27 (C-7), 156.68 (C-8a), 152.95 (C-5), 136.15 (C-2′), 135.96 (C-1′), 130.82 (C-3′), 128.23, 127.35, 126.80 (C-4′, C-5′, C-6′), 101.51 (C-6), 59.38 (O*C*H_2_CH_3_), 47.95 (C-2), 41.59 (C-3), 17.36 (2-CH_3_), 14.12 (OCH_2_ *C*H_3_) ppm; MS (EI): M^+·^ C_16_H_18_N_4_O_2_^+·^ calc. (Et-ester) 298.1430, found 298.1429, calc. (Me-ester) 284.1174, found 284.1170; *m/z* (%): Et-ester: M^+·^ 298 (100), [M–H]^+^ 297 (44), Me-ester: M^+·^ 284 (39), [M–H]^+^ 283 (25), [M–Et]^+^ and [M–Me]^+^ 269 (8), [M–OEt]^+^ and [M–OMe]^+^ 253 (25), [M–HOEt]^+·^ and [M–HOMe]^+·^ 252 (11), [M–H–HOEt]^+^ and [M–H–HOMe]^+^ 251 (35), [M–C_2_H_4_OOC]^+·^ and [M–H_2_COOC]^+·^ 226 (70), [M–H–C_2_H_4_OOC]^+^ and [M–H–H_2_COOC]^+^ 225 (27), 158 (61), 131 (10), 131 (12), 120 (18), 118 (32), 117 (18), 91 (48), 77 (15), 65 (28).

#### *Ethyl (methyl) 5*-*imino*-*1*-*(4*-*methylphenyl)*-*2,3*-*dihydroimidazo[1,2*-*a]pyrimidine*-*6*-*carboxylate* (**7f**, C_16_H_18_N_4_O_2_)

According to general method with 2.51 g of **5f**. The method afforded 0.43 g (29 %) of **7f** (a 68:32 mixture of the Et- and Me-esters based on the intensity of the CH_2_-signals in the NMR spectrum. The abundance ratio of the M^+·^ ions was 73:27). M.p.: 140–142 °C; *R*_*f*_ = 0.00 (toluene/ethyl acetate/methanol 1:3:0.5); NMR data for **7f**: ^1^H NMR (500 MHz, DMSO-*d*_6_): *δ* = 8.15 (s, 1H, H-7), 8.09 (br s, 1H, 5-N*H*), 7.60 (m, 2H, H-2′, H-6′), 7.22 (m, 2H, H-3′, H-5′), 4.19 (m, 2H, H-2), 4.19 (q, 2H, *J* = 7.1 Hz, OC*H*_*2*_CH_3_), 2.30 (s, 3H, 4′-CH_3_), 1.26 (t, 3H, *J* = 7.1 Hz, OCH_2_C*H*_3_) ppm; ^13^C NMR (125 MHz, DMSO-*d*_6_): *δ* = 165.28 (C=O), 157.56 (C-7), 155.31 (C-8a), 152.72 (C-5), 135.82 (C-1′), 133.51 (C-4′), 129.11 (C-3′, C-5′), 120.11 (C-2′, C-6′), 102.39 (C-6), 59.54 (O*C*H_2_CH_3_), 45.84 (C-2), 40.57 (C-3), 20.31 (4′-CH_3_), 14.08 (OCH_2_ *C*H_3_) ppm; MS (EI): M^+·^ C_16_H_18_N_4_O_2_^+·^ calc. (Et-ester) 298.1430, found 298.1426, calc. (Me-ester) 284.1284, found 284.1279; *m/z* (%): Et-ester: M^+·^ 298 (61), [M–H]^+^ 297 (10), Me-ester: M^+·^ 284 (22), [M–H]^+^ 283 (7), [M–OEt]^+^ and [M–OMe]^+^ 253 (29), [M–HOEt]^+·^ and [M–HOMe]^+·^ 252 (14), [M–H–HOEt]^+^ and [M–H–HOMe]^+^ 251 (29), [M–C_2_H_4_OOC]^+·^ and [M–H_2_COOC]^+·^ 226 (100), [M–H–C_2_H_4_OOC]^+^ and [M–H–H_2_COOC]^+^ 225 (26), 120 (34), 118 (14), 91 (39), 77 (10), 65 (17).

#### *Ethyl (methyl) 5*-*imino*-*1*-*(2*-*methoxyphenyl)*-*2,3*-*dihydroimidazo[1,2*-*a]pyrimidine*-*6*-*carboxylate* (**7g**, C_16_H_18_N_4_O_3_)

According to general method with 2.67 g of **5** **g**. The method afforded 0.30 g (19 %) of **7** **g** (a 68:32 mixture of the Et- and Me-esters based on the intensity of the CH_2_-signals in the NMR spectrum. The abundance ratio of the M^+·^ ions was 67:33). M.p.: 165–168 °C; *R*_*f*_ = 0.00 (toluene/ethyl acetate/methanol 1:3:0.5); NMR data for **7** **g**: ^1^H NMR (500 MHz, DMSO-*d*_6_): *δ* = 8.08 (br s, 1H, 5-N*H*), 8.02 (s, 1H, H-7), 7.38 (m, 1H, H-6′), 7.36 (m, 1H, H-4′), 7.16 (d, 1H, *J* = 7.8 Hz, H-3′), 7.01 (dt, 1H, *J* = 1.2 Hz, 7.6 Hz, H-5′), 4.17 (q, 2H, *J* = 7.1 Hz, OC*H*_*2*_CH_3_), 4.06 (m, 2H, H-2), 4.06 (m, 2H, H-3), 3.82 (s, 3H, 2′-OCH_3_), 1.20 (t, 3H, *J* = 7.1 Hz, OCH_2_C*H*_3_) ppm; ^13^C NMR (125 MHz, DMSO-*d*_6_): *δ* = 165.39 (C=O), 158.26 (C-7), 157.26 (C-8a), 154.90 (C-2′), 152.86 (C-5), 129.35, 128.98 (C-4′, C-6′), 125.50 (C-1′), 120.46 (C-5′), 112.48 (C-3′), 101.57 (C-6), 59.37 (O*C*H_2_CH_3_), 55.66 (2′-OCH_3_), 47.26 (C-2), 41.50 (C-3), 14.11 (OCH_2_ *C*H_3_) ppm; MS (EI): M^+·^ C_16_H_18_N_4_O_2_^+·^ calc. (Et-ester) 314.1379, found 314.1377, calc. (Me-ester) 300.1223, found 300.1225; *m/z* (%): Et-ester: M^+·^ 314 (100), [M–H]^+^ 313 (17), Me-ester: M^+·^ 300 (50), [M–H]^+^ 299 (11), [M–OMe]^+^ 283 (29), [M–OEt]^+·^ and [M–OMe]^+·^ 269 (47), [M–H–HOEt]^+^ and [M–H–HOMe]^+^ 267 (17), [M–C_2_H_4_OOC]^+·^ and [M–H_2_COOC]^+·^ 242 (71), [M–H–C_2_H_4_OOC]^+^ and [M–H–H_2_COOC]^+^ 241 (11), 237 (17), 212 (14), 211 (17), 174 (34), 134 (10), 119 (11), 106 (6), 105 (5), 92 (6), 77 (8), 65 (6).

#### *Ethyl (methyl) 5*-*imino*-*1*-*(4*-*methoxyphenyl)*-*2,3*-*dihydroimidazo[1,2*-*a]pyrimidine*-*6*-*carboxylate* (**7h**, C_16_H_18_N_4_O_3_)

According to general method with 2.67 g of **5** **h**. The method afforded 0.53 g (34 %) of **7** **h** (a 85:15 mixture of the Et- and Me-esters based on the intensity of the CH_2_-signals in the NMR spectrum. The abundance ratio of the M^+·^ ions was 88:12). M.p.: 181–182 °C; *R*_*f*_ = 0.00 (toluene/ethyl acetate/methanol 1:3:0.5); NMR data for **7** **h**: ^1^H NMR (500 MHz, DMSO-*d*_6_): *δ* = 8.13 (s, 1H, H-7), 8.08 (br s, 1H, 5-N*H*), 7.59 (dd, 2H, *J* = 2.4 Hz, 6.9 Hz, H-2′, H-6′), 6.99 (dd, 2H, *J* = 2.3 Hz, 6.9 Hz, H-3′, H-5′), 4.18 (m, 2H, H-2), 4.18 (q, 2H, *J* = 7.1 Hz, OC*H*_*2*_CH_3_), 4.02 (m, 2H, H-3), 3.76 (s, 3H, 4′-OCH_3_), 1.25 (t, 3H, *J* = 7.1 Hz, OCH_2_C*H*_3_) ppm; ^13^C NMR (125 MHz, DMSO-*d*_6_): *δ* = 165.32 (C=O), 157.71 (C-7), 156.28 (C-4′), 155.42 (C-8a), 152.78 (C-5), 131.21 (C-1′), 122.34 (C-2′, C-6′), 113.91 (C-3′, C-5′), 102.07 (C-6), 59.49 (O*C*H_2_CH_3_), 55.21 (4′-OCH_3_), 46.33 (C-2), 40.62 (C-3), 14.09 ppm (OCH_2_ *C*H_3_); MS (EI): M^+·^ C_16_H_18_N_4_O_2_^+·^ calc. (Et-ester) 314.1379, found 314.1374, calc. (Me-ester) 300.1223, found 300.1219; *m/z* (%): Et-ester: M^+·^ 314 (100), [M–H]^+^ 313 (22), Me-ester: M^+·^ 300 (13), [M–H]^+^ 299 (6), [M–OEt]^+·^ and [M–OMe]^+·^ 269 (21), [M–HOEt]^+^ and [M–HOMe]^+^ 268 (12), [M–H–HOEt]^+^ and [M–H–HOMe]^+^ 267 (32), [M–C_2_H_4_OOC]^+·^ and [M–H_2_COOC]^+·^ 242 (87), [M–H–C_2_H_4_OOC]^+^ and [M–H–H_2_COOC]^+^ 241 (21), 136 (30), 134 (10), 121 (10), 77 (5).

## Footnotes

1 PERCH Solutions Ltd. http://perchsolutions.com
